# RAB23 regulates musculoskeletal development and patterning

**DOI:** 10.3389/fcell.2023.1049131

**Published:** 2023-02-23

**Authors:** Md. Rakibul Hasan, Anna Koskenranta, Kirsi Alakurtti, Maarit Takatalo, David P. Rice

**Affiliations:** ^1^ Orthodontics, Oral and Maxillofacial Diseases, University of Helsinki, Helsinki, Finland; ^2^ Helsinki University Hospital, Helsinki, Finland

**Keywords:** knee, patella, RAB23, TGFβ2, scleraxis, GLI1, tenogenesis

## Abstract

RAB23 is a small GTPase which functions at the plasma membrane to regulate growth factor signaling. Mutations in *RAB23* cause Carpenter syndrome, a condition that affects normal organogenesis and patterning. In this study, we investigate the role of RAB23 in musculoskeletal development and show that it is required for patella bone formation and for the maintenance of tendon progenitors. The patella is the largest sesamoid bone in mammals and plays a critical role during movement by providing structural and mechanical support to the knee. *Rab23* ^*−/−*^ mice fail to form a patella and normal knee joint. The patella is formed from *Sox9* and *scleraxis (Scx)* double-positive chondroprogenitor cells. We show that RAB23 is required for the specification of SOX9 and scleraxis double-positive patella chondroprogenitors during the formation of patella anlagen and the subsequent establishment of patellofemoral joint. We find that scleraxis and SOX9 expression are disrupted in *Rab23* ^*−/−*^ mice, and as a result, development of the quadriceps tendons, cruciate ligaments, patella tendons, and entheses is either abnormal or lost. TGFβ-BMP signaling is known to regulate patella initiation and patella progenitor differentiation and growth. We find that the expression of TGFβR2, BMPR1, BMP4, and pSmad are barely detectable in the future patella site and in the rudimentary tendons and ligaments around the patellofemoral joint in *Rab23* ^*−/−*^ mice. Also, we show that GLI1, SOX9, and scleraxis, which regulate entheses establishment and maturation, are weakly expressed in *Rab23* ^*−/−*^ mice. Further analysis of the skeletal phenotype of *Rab23* ^*−/−*^ mice showed a close resemblance to that of *Tgfβ2* ^*−/−*^ mice, highlighting a possible role for RAB23 in regulating TGFβ superfamily signaling.

## Introduction

Ras-associated protein 23 (RAB23) belongs to the large family of RAB GTPase proteins, many of which are key regulators of intracellular membrane trafficking events (Stenmark, 2009). They control intercellular signaling by restricting ligand secretion and by cargo (ligand-bound receptor) internalization (Entchev et al., 2000; Lanzetti et al., 2000). RAB23 is localized at the plasma membrane and is proposed to be involved in the endocytic pathway (Evans et al., 2003).

RAB23 regulates growth factor signaling (Eggenschwiler et al., 2001; Fuller et al., 2014; Hasan et al., 2020), and mutations in *RAB23* affect the development and patterning of multiple organs and give rise to a diverse complex congenital anomaly, collectively known as Carpenter syndrome (CS, MIM# 201000) or acrocephalopolysyndactyly type II (Carpenter, 1909; Jenkins et al., 2007). CS causes a wide spectrum of defects, such as craniosynostosis, congenital heart defects, obesity, polydactyly, and multiple skeletal abnormalities including decreased hip mobility, spina bifida occulta, kyphoscoliosis, short muscular neck, genu valgum, and lateral displacement of the patella and mandibular defects (Cohen et al., 1987; Jenkins et al., 2007). Rab23 *open brain* mutant mice exhibit abnormalities in the neural tube, vertebral column, and axial skeleton, such as the ribs, limbs, and skull (Gunther et al., 1994; Eggenschwiler and Anderson, 2000; Hasan et al., 2020).

RAB23 acts as an inhibitor of the Hedgehog (Hh) pathway with RAB23 possibly restricting the entry of Smo (smoothened) to the cilium (Eggenschwiler et al., 2001; Boehlke et al., 2010). However, the direct ciliary function appears unlikely as endogenous RAB23 has not been localized to the primary cilium but to the plasma membrane and the endocytic pathway, and additionally, cilium length and function are reported to be normal in *Rab23* ^*opb2*^ nodes (Evans et al., 2003; Fuller et al., 2014). RAB23 is also reported to have Hh pathway-independent functions in left–right asymmetry (Fuller et al., 2014) and in the limb, neural tube, and cranial facial development (Eggenschwiler et al., 2006). *Rab23* ^*opb2*^ mutations caused lateralization defects linked to defective nodal signal production in the lateral plate mesoderm (Fuller et al., 2014). We have previously demonstrated that RAB23 regulates early calvarial osteogenesis in mice through canonical FGFR signaling and non-canonical Hh signaling (Hasan et al., 2020).

The patella, commonly known as the kneecap, is the largest sesamoid bone in mammals. Like other sesamoid bones, the patella bone is flat, located close to a joint, and embedded within tendons (Samuels et al., 2017; Eyal et al., 2019). The patella plays an important role during movement, providing structural support and stability to the knee joint (Lennox et al., 1994; Sarin et al., 1999). Relatively little is known about the development of the patella bone. Mouse studies showed that the patella initially develops as a part of the femur from *scleraxis (Scx)* and *Sox9* double-positive chondroprogenitor cells (Blitz et al., 2013; Eyal et al., 2015). TGFβ2 signaling is required for the specification of the patella chondroprogenitor cells, and subsequently BMP signaling is required for the differentiation and growth of the patella progenitors to make the patella anlagen. The patella anlagen separate from the femur by making the patellofemoral synovial joint (Eyal et al., 2015; Eyal et al., 2019). Even though RAB23 is known to regulate aspects of musculoskeletal patterning and development, how RAB23 contributes to patella development has not been studied.

Here, we show that RAB23-deficient mice failed to develop patella and a loss of Scx expression in patella progenitor cells. SOX9 was initially expressed in the patella progenitors, but this expression was transient and lost at later developmental stages. Concomitant to Scx and SOX9 disruption, TGFβ-BMP signaling, as well as phospho-pErk44/42 and GLI1, also affected the patella and surrounding musculoskeletal structures. These anomalies collectively rendered a lack of patella anlagen and disruption of the tendons, ligaments, and entheses of the knee joint. We further analyzed the skeletal phenotype of *Rab23* ^*−/−*^ mice and found that the overall phenotype closely resembles that of TGFβ2-null mice, which may indicate a role for RAB23 in regulating TGFβ superfamily signaling.

## Materials and methods

### Mice

The null allele mice with *Rab23* ^*−/−*^ mutation was originally in C3H/Hej background backcrossed to the C57Bl/6J mouse strain (Charles River). The genotype of all mice and embryos were verified with PCR-based genotyping as previously described (Kasarskis et al., 1998; Eggenschwiler and Anderson, 2000). *Rab23* ^*−/−*^ mice survive until embryonic days E18.5 and die neonatally. The details of the *Rab23* ^*−/−*^ mouse generation could be found as explained (Hasan et al., 2020).

## Antibodies and reagents

Rabbit polyclonal anti-RAB23 antibody (Cat#11101-1-AP) was purchased from Proteintech. Rabbit polyclonal anti-SOX9 antibody (Cat#AB5535) was purchased from Sigma-Aldrich. Rabbit polyclonal anti-BMPR1A antibody (Cat#38-6000) was purchased from Invitrogen. Rabbit polyclonal anti-pSmad1/5/8 (Cat#AB3848) was purchased from Millipore. Rabbit monoclonal anti-phospho-p44/42 (Cat#9101) was purchased from Cell Signaling Technology. Mouse monoclonal anti-BMP4 (SC-12721), monoclonal anti-Tenascin-C (E-9, SC-25328), monoclonal anti-GDF-5 (A-10, SC-373744), monoclonal anti-GLI-1 (C-1, SC-515751), monoclonal anti-TGFβR2 (D2, SC-17799), monoclonal anti-COL1A1 (3G3, SC-293182), monoclonal anti-COL2A1 (M2138, SC-52658), and monoclonal anti-Scleraxis (A-7, SC-518082) antibodies were purchased from Santa Cruz Biotechnology. Goat anti-rabbit IgG (H + L) Alexa 488 (Cat#A-11008), goat anti-mouse IgG (H + L) Alexa 546 (Cat#A-110003), goat anti-mouse IgG (H + L) Alexa 488 (Cat#A-11001), and goat anti-rabbit IgG (H + L) Alexa 546 (Cat#A11010) were purchased from Thermo Fisher Scientific. Anti-Digoxigenin-AP, Fab fragments (Cat#11093274910), and BM purple AP substrate precipitating (Cat#11442074001) were purchased from Roche. 4% PFA in PBS (Cat#15424389) and Hoechst 33342 (Cat# H3570) were purchased from Thermo Fisher Scientific. BSA (Cat#A3059) and Triton X-100 (Cat#T8787) were purchased from Sigma-Aldrich. EdU (5-ethynyl-2′-deoxyuridine) (reference number: A10044) and the Click-iT EdU Alexa Fluor 594 Imaging kit (reference number: C10339) were purchased from Invitrogen.

### *In situ* hybridization

For *in situ* hybridization, Wt and *Rab23* ^*−/−*^ embryos were, respectively, collected at E14.0 and E15.5, dissected and fixed in 4% paraformaldehyde (PFA) overnight, followed by dehydration up to 100% ethanol, and then, embedded in paraffin. The knee samples were sagittally sectioned (7 μm) for digoxigenin label *in situ* hybridization. *In situ* hybridization was performed according to a standard protocol. For the detection of digoxigenin-labeled RNA probes, BMP purple AP substrate precipitating solution (Roche) was used. The following probes were used: *Sox9*, *Scx*, and *Gli1* (Thomas et al., 1997; Schweitzer et al., 2001; Veistinen et al., 2017). All probes were transcribed from plasmids.

### Immunohistochemistry

Immunohistochemistry on the sagittal sections (7 μm) of the knee was performed using RAB23, SOX9, Scleraxis, Tenascin-C, GDF5, GLI1, TGFβR2, BMPR1, BMP4, pSmad1/5/8, phospho-p44/42, COL1A1, and COL2A1 primary antibodies. Alexa fluor 488 and Alexa fluor 546 secondary antibodies were used to detect the primary antibodies. The samples were permeabilized by triton X-100 and antigen retrieval was performed by citrate buffer. All the other procedures were followed according to the protocol described (Veistinen et al., 2017; Hasan et al., 2020).

### Immunofluorescence microscopy

Fluorescence microscopy was performed at the Biomedicum imaging unit, University of Helsinki. All the immunohistochemical samples were imaged using a Zeiss Axio Imager Z2 microscope. Excitation was achieved using 350/488/594 nm wavelength. All images were taken at RT and analyzed with Fiji ImageJ 1.51b (64-bit) software.

### Skeletal staining

The Alcian blue–Alizarin red staining of E18.5 whole body was performed as previously described (Rice et al., 2003). Sectional tissue samples were stained with Alcian blue and nuclear fast red. For the analysis of bone, cartilage and sectional tissue images were captured using Analysis software (Soft Imaging System) and Olympus BX41 microscope and analyzed using Image J.

### EdU pulsing and staining

Cell proliferation was assessed by prior EdU incorporation followed by the EdU click reaction. The EdU was detected with the Click-iT EdU imaging kit, Alexa fluor 594 (Molecular probes, Invitrogen), according to the manufacturer’s instruction.

The mice were pulsed with 0.05 mg/g (i.p.) EdU in PBS for 2 h. After sacrificing E15.5 and E17.5, embryonic hind limbs were collected and fixed o/n with 4% paraformaldehyde and processed for paraffin sections. Paraffin-embedded Wt and *Rab23* ^*−/−*^ embryonic hind limbs were sectioned sagittally with 7 μm size. The sections were deparaffinized and stained with a Click-iT kit. During the EdU click reaction procedure, in brief, sectional explants were deparaffinized by xylene and rehydrated by a gradient of ethanol series. After washing with 2 mg/mL glycine, limb sections were permeabilized with 0.5% Triton X-100. After washing several times in PBS, 10 μM EdU cocktail was used for click reaction for 30 min in the dark room. After PBS washing several times, 5 μg/mL Hoechst was used for counterstaining. The Vectashield mounting medium was used to mount the slides. Slides were then imaged with a fluorescence microscope with an emission wavelength of 615 nm.

Cell counting: The number of EdU-positive cells and total cells was counted from the end of femur head (289 × 96 pixels), Wt patella tendon *vs. Rab23* ^*−/−*^ patella tendon-like structure, and Wt quadriceps tendon *vs. Rab23* ^*−/−*^ tendon-like structure (143 × 144 pixels) at the embryonic stage E15.5 and E17.5. The total cell number was determined with Hoechst 33342 staining of nuclei.

### *In situ* apoptosis detection

*In situ* apoptosis detection assay on the hind limb sagittal sections at E14.0 and E15.5 in Wt and *Rab23* ^*−/−*^ mice was performed according to the manufacturer’s instruction (Abcam, ab206386).

### X-ray micro-computed tomography

E18.5 WT and *Rab23* ^*−/−*^ whole-body and limb samples were collected and fixed with 4% paraformaldehyde overnight. The whole body, excluding limb samples, was dehydrated by gradient ethanol series to 70% (v/v), and limb samples were additionally stained with 0.3% phosphotungstic acid (PTA) in 70% ethanol followed by both samples being processed for x-ray microtomography (μCT) imaging with Bruker SkyScan1272 (desktop micro-CT system, Bruker microCT N.V., Kontich, Belgium). Tomography 3D reconstructions were obtained using the program NRecon (desktop micro-CT system, Bruker microCT N.V., Kontich, Belgium).

### Statistical analysis

Paired Student’s t-test has been applied to perform the statistics of all the data obtained from EdU experiment and skeletal measurements. *p* value less than 0.05 is considered statistically significant.

## Results

### RAB23-deficient mice fail to develop patella

**FIGURE 1:**
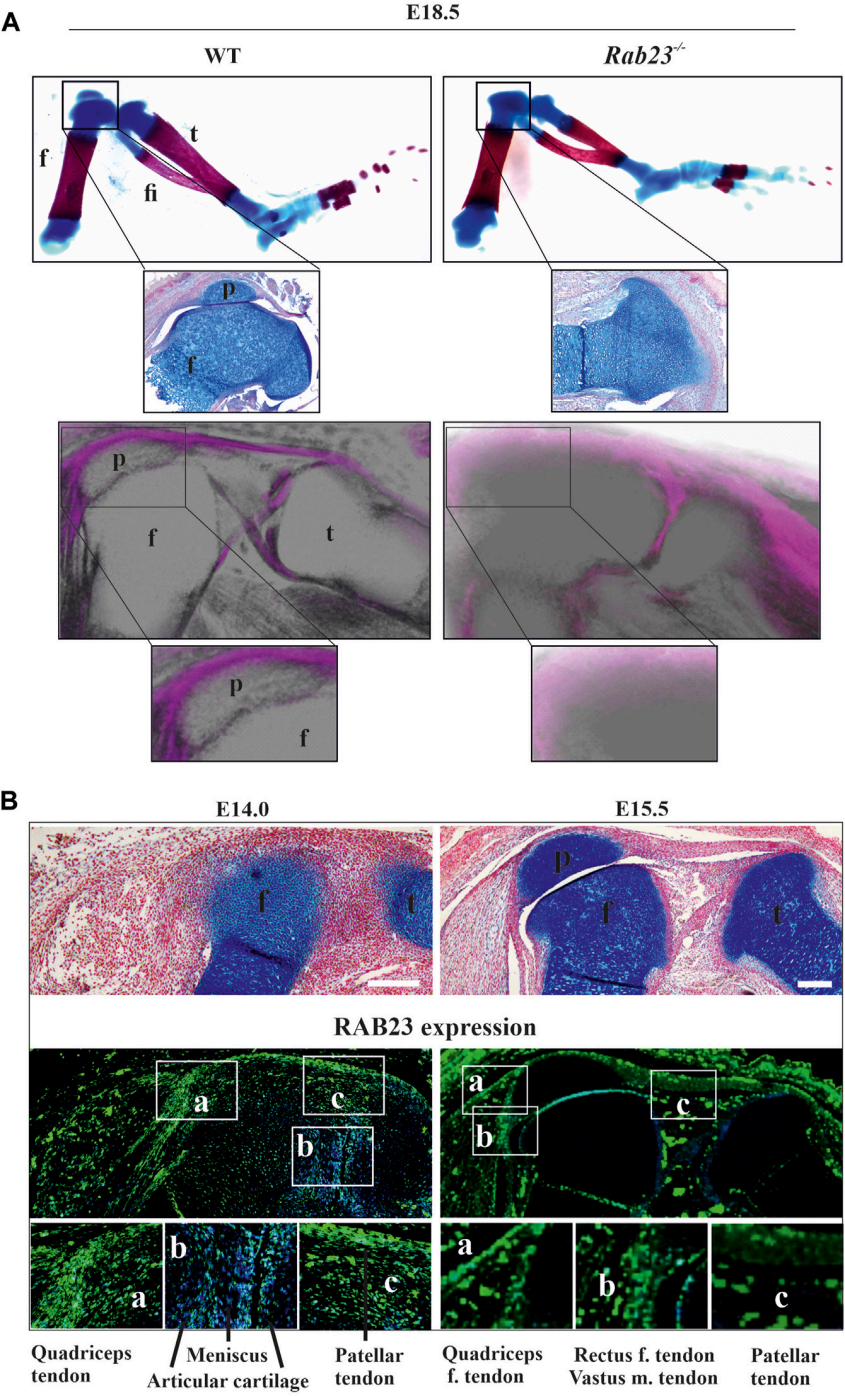
*Rab23* ^*−/−*^ mice failed to develop patella and analysis of RAB23 protein expression in the developing patella. **(A)** Analysis of patella formation in Wt and *Rab23* ^*−/−*^ mice at E18.5 by Alcian blue and Alizarin red staining (upper panel) and by µ-CT imaging (lower panel). Both analysis methods show that while Wt mice developed patella, *Rab23* ^*−/−*^ mice failed to form patella. The absence of patella development in *Rab23* ^*−/−*^ mice was further confirmed by Alcian blue and fast red staining of the sagittal-sectional tissue from the hind limbs (inset, upper panel) at E18.5. (*n* = 10 samples for each genotype). p: patella, f: femur, t: tibia, and fi: fibula. **(B)** Immunohistochemical expression analysis of RAB23 in the Wt sagittal-sectional tissues of the hind limbs obtained from E14.0 and E15.5 embryos (lower panel). Expression analysis shows that RAB23 is expressed in the developing patella, patella tendon, quadriceps tendon, rectus femoris tendon, and vastus medialis tendon. RAB23 expression is also observed in the meniscus and articular cartilage. To highlight the cartilage cells of the patella, sagittal sections from E14.0 and E15.5 hind limbs are stained with Alcian blue and fast red (upper panel). (*n* = 3 samples for each age). p: patella, f: femur, and t: tibia. Scale bar: 200 μm.

Skeletal analysis by Alcian blue and Alizarin red and also by μ-CT of hind limbs in Wt and *Rab23* ^*−/−*^ mice at E18.5 showed that *Rab23* ^*−/−*^ mice failed to develop the patella (Figure 1A). We further analyzed this developmental defect in sagittal tissue sections stained with Alcian blue staining of the patellofemoral sagittal sectional tissues at E18.5 (Figure 1A, inset, upper panel). To understand if the failure of patella formation in *Rab23* ^*−/−*^ mice was due to the programmed cell death of patella chondroprogenitors, we performed *in situ* apoptosis detection assay on the hind limb sagittal sections at E14.0 and E15.5 in Wt and *Rab23* ^*−/−*^ mice. The results show that the failure of patella formation in *Rab23* ^*−/−*^ mice were not due to cell death (Supplementary Figure S1).

### RAB23 expression analysis in the developing patella

To understand the expression of RAB23 in the developing patella, we performed immunohistochemical (IHC) staining on the sagittal sections of hind limbs obtained from E14.0 and E15.5 WT mice. We found that RAB23 was highly expressed in the future patella region at E14.0 (Figure 1B). At both E14.0 and E15.5 developmental stages, RAB23 is widely expressed in the patellar tendon, cruciate ligaments, in the meniscus, articular cartilage, quadriceps tendon and muscles, rectus femoris tendon, and vastus medialis tendon (Figure 1B). We have validated the specificity of RAB23 antibody by IHC staining on the sagittal sections of hind limbs obtained from E14.0 and E15.5 *Rab23* ^*−/−*^ mice (Supplementary Figure S2).

### Scleraxis and SOX9 expression are affected in the patella musculoskeletal structure in *Rab23* ^*−/−*^ mice

**FIGURE 2:**
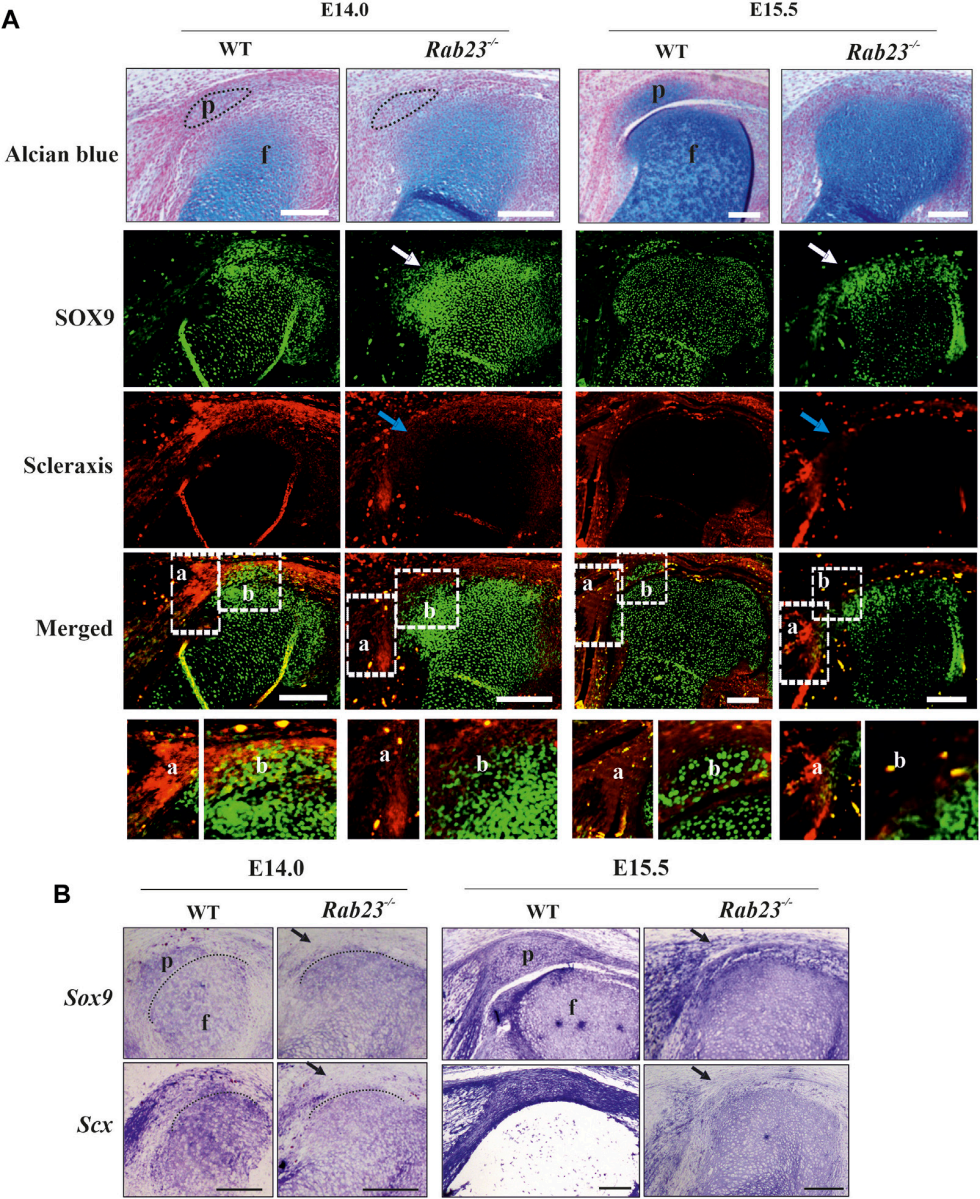
RAB23 deficiency failed to specify Scx and SOX9 double-positive patella progenitor cells. **(A, B)** Immunohistochemical **(A)** and *in situ* **(B)** expression analysis of Scleraxis (Scx) and SOX9 in the Wt and *Rab23* ^*−/−*^ mice sagittal-sections of the hind limbs obtained from E14.0 and E15.5 embryos. Wt samples show the expression of Scx and SOX9 double-positive patella progenitor cells, while *Rab23* ^*−/−*^ samples fail to express Scx and SOX9 double-positive progenitor cells **(A)**. In Wt samples, Scx shows robust expression in the future patella site (E14.0) and shows expression in the patella tendon, quadriceps tendon, rectus femoris, and vastus medialis tendon **(A, B)**. Scx expression in *Rab23* ^*−/−*^ samples is missing in the future patella site at E14.0 (blue arrow) and is barely detectable in the malformed patella tendon-like structure and quadriceps tendon **(A, B)**. At E15.5 in *Rab23* ^*−/−*^ samples, Scx remained undetectable at the patella site (blue arrow and black arrow) and shows rudimentary expression in the patella tendon and in the quadriceps tendon **(A, B)**. Analysis of SOX9 expression shows that in Wt samples, SOX9 was expressed in the future patella site at E14.0 and in the patella at E15.5 **(A, B)**. In *Rab23* ^*−/−*^ samples, SOX9 was initially barely expressed in the future patella site at E14.0 (white arrow and black arrow) but was abolished at E15.5 samples (white arrow) **(A)**. While SOX9 showed uniform expression throughout the articular cartilage in the Wt femur toward the patella and tibia, *Rab23* ^*−/−*^ samples showed more SOX9 expressions in the femur head toward the imaginary patellofemoral site **(A, B)**. To highlight the cartilage cells of the patella, sagittal sections from E14.0 and E15.5 hind limbs were stained with Alcian blue and fast red (A, upper panel). Black dotted oval shape indicates the future patella site **(A)**. Black dotted marking indicates the end of the femur toward the patella **(B)** (*n* = 3 samples for each age and genotype). p: patella and f: femur. Scale bar: 200 μm.

To understand how RAB23 influences patella development, we examined the expression and co-expression of SOX9 and Scx in chondroprogenitor cells in Wt and *Rab23* ^*−/−*^ mice (Figure 2). Co-expression analysis by IHC staining revealed that RAB23 deficiency caused the ablation of patella progenitor cells (Figure 2A). Wt controls showed SOX9 and Scx double-positive patella progenitor cells at E14.0, and the patella already developed at E15.5 were about to separate from the femur (Figure 2A).

Detailed analysis by IHC and by *in situ* hybridization showed that Scx expression in the future patella site was lost and was poorly detectable in the rectus femoris tendon and vastus medialis tendon and in the patellar tendon in *Rab23* ^*−/−*^ samples at E14.0 (Figures 2A, B). At E15.5, Scx was not detectable in the future patella site but showed disorganized expression in the patella tendon-like structure and rectus femoris tendon and vastus medialis tendon in *Rab23* ^*−/−*^ samples (Figures 2A, B). However, in Wt samples, Scx showed robust expression in the patella site, in the patella tendon, in the rectus femoris tendon, and vastus medialis tendon (Figures 2A, B). In addition, we stained Wt and *Rab23* ^*−/−*^ samples with COL1A1 at E15.5 and E17.5 to understand whether the disrupted expression of Scx in *Rab23* ^*−/−*^ samples could show an effect on subsequent maturation of rectus femoris tendon and vastus medialis tendon. The results showed that COL1A1 in *Rab23* ^*−/−*^ samples were expressed poorly and failed to mark these tendon structures at both the embryonic stage, while Wt samples showed a well-formed structure of these tendons (Supplementary Figure S3). Interestingly, the chondroprogenitor marker SOX9 very well marked the future patella and the boundary of articular cartilage of the femur in Wt samples, and in *Rab23* ^*−/−*^ samples, SOX9 was poorly expressed in the patella site at E14.0 and lost at E15.5 (Figure 2). SOX9 was misexpressed in these samples and heterogeneously marked the boundary of the articular cartilage of the femur (Figure 2). The expression of SOX9 was more prominent toward the femur head in *Rab23* ^*−/−*^ samples (Figure 2). We further stained E15.5 and E17.5 Wt and *Rab23* ^*−/−*^ samples with COL2A1, a marker for chondrocytes. We found that consistent with SOX9 expression, COL2A1 showed an expanded expression in the articular cartilage toward the femur and tibia heads in *Rab23* ^*−/−*^ samples (Supplementary Figure S4). Collectively, these data indicated that RAB23 is required for the specification of patella progenitor cells where RAB23 regulates the co-expression of scleraxis and SOX9 in the patella chondroprogenitor for patella anlagen formation. As Scx along with COL1A1 is the earliest marker for tendon and ligament formation, RAB23 regulation of Scx and COL1A1 suggests a role for RAB23 in knee joint tendonogenesis and ligamentogenesis.

### Cell proliferation in the developing patella in Wt and *Rab23* ^*−/−*^ mice

**FIGURE 3:**
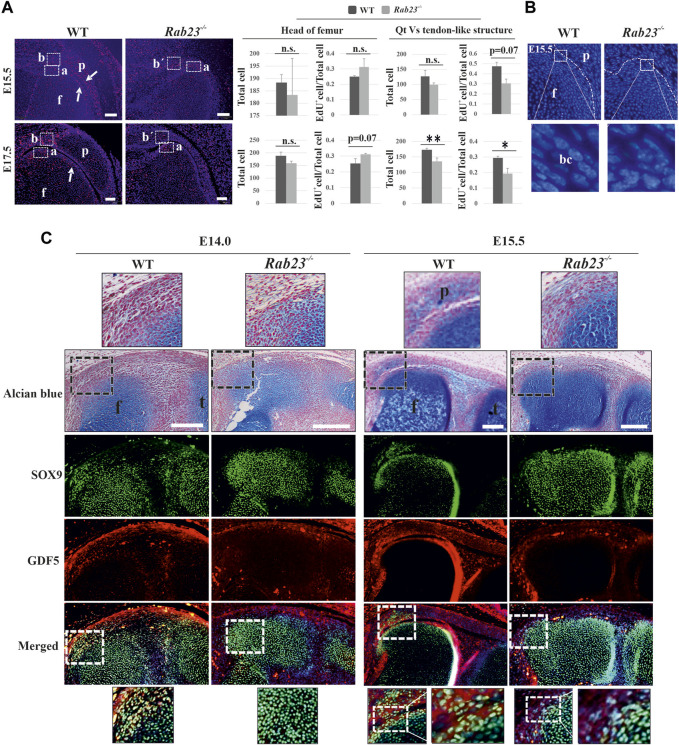
Analysis of cell proliferation and the expression of GDF5 in the Wt and *Rab23* ^*−/−*^ patellofemoral samples. **(A)** EdU pulsed cell proliferation analysis in the patellofemoral Wt and *Rab23* ^*−/−*^ samples at E15.5 and E17.5. Images show during and after the separation of the patella from the femur, the boundary cells undergo robust cell proliferation in Wt samples at E15.5 and E17.5, (white arrows). On the contrary, no patella and patellofemoral joint were established in *Rab23* ^*−/−*^ samples, but showed an expansion of the femur head toward the presumptive patella site. We, therefore, compared cell proliferation at the end of the femur **(A)** in Wt and *Rab23* ^*−/−*^ samples and also compared the cell proliferation of quadriceps tendon **(B)** in Wt *vs.* tendon-like structure (b´) in *Rab23* ^*−/−*^ samples at E15.5 and E17.5. Counting of all cells (blue + EdU^*+*^ cells) and the ratio of EdU^*+*^ cells (EdU^*+*^ cells/all cells) show that proliferative cells in the femur head increased non-significantly in *Rab23* ^*−/−*^ compared to Wt at E15.5 and E17.5. Counting of all cells (blue + EdU^*+*^ cells) and the ratio of EdU^*+*^ cells (EdU^*+*^ cells/all cells) show that proliferative cells in the tendon-like structure decreased significantly in *Rab23* ^*−/−*^ compared to Wt at E15.5 and E17.5 (*n* = 3 samples for each genotype). *p* value ˂ 0.05 (*); *p* value ˂ 0.02 (**). Nuclei were stained with Hoechst (blue), EdU^*+*^ cells (red), Qt: quadriceps tendon, p: patella, and f: femur. Scale bar: 100 μm. **(B)** Morphological observation of boundary cells of the femur toward the patella in Wt *vs.* boundary cells of the femur in *Rab23* ^*−/−*^ at E15.5. Images show the cell nuclear shape in the boundary cells is flat and elongated in Wt samples (inset), whereas *Rab23* ^*−/−*^ samples show the cell nuclear shape is morphologically irregular (inset). Nuclei (blue), bc: boundary cell. **(C)** Immunohistochemical analysis of GDF5 and SOX9 in Wt and *Rab23* ^*−/−*^ mice sagittal-sectional tissues of the hind limbs at E14.0 and E15.5. Wt samples at E14.0 show GDF5 expression in the interzone cells between the future patella and femur junction where GDF5 mostly co-localizes with SOX9. At E15.5, GDF5 is expressed at the femur and patella periphery closer to the patellofemoral joint. GDF5 is not expressed in *Rab23* ^*−/−*^ samples at E14.0 and shows poor expression in the femur head at E15.5 *Rab23* ^*−/−*^ samples. To highlight the cartilage cells of the patella, femur and interzone cell sagittal sections from E14.0 and E15.5 hind limbs were stained with Alcian blue and fast red (upper panel). (*n* = 3 samples for each age and genotype). p: patella, f: femur, and t: tibia. Scale bar: 200 μm.

To understand cell proliferation during the development of patella, EdU pulsed Wt and *Rab23* ^*−/−*^ mice knee samples were analyzed at E15.5 and E17.5, respectively. The results showed that the cells in the patella, in the quadriceps tendon, and in the boundary cell in Wt samples were highly proliferative (Figure 3A). On the contrary, *Rab23* ^*−/−*^ samples failed to develop a patella and it showed more proliferating cells toward the femur head where SOX9 and COL2A1 expression have been expanded (Figure 3A and Supplementary Figure S4). Sequential tissue sections of Wt knee samples revealed that during patella development, the joint-forming cells initially undergo robust proliferation at the interzone (data not shown) and later when the patellofemoral joint has been established remained proliferative at the vicinity of the patellofemoral joint (Figure 3A). No such patellofemoral joint was found in *Rab23* ^*−/−*^ samples and instead of the quadriceps tendon, a malformed tendon-like structure was observed which represented reduced and aberrant EdU pulsed proliferative cells (Figure 3A). Here, we observed that the nuclear morphology of the interzone or boundary cells was flat and elongated in the Wt sample at E15.5. As *Rab23* ^*−/−*^ mice failed to develop patella, no such interzone or boundary was observed. Therefore, the cells on the tip of the femur were morphologically irregular and showed different shapes (Figure 3B).

### Analysis of GDF5 expression in Wt and *Rab23* ^*−/−*^ boundary cells during patellofemoral joint formation

While the patella anlagen tend to separate from the femur by joint formation, joint-forming interzone (zone between the femur and patella anlagen) cells gradually lose their chondrocyte appearance and start the expression of *Gdf5*, *Tapp3*, *Wnt9a*, and *Wnt4* as boundary marking genes (Eyal et al., 2015). To understand whether RAB23 influenced the expression of GDF5 that marked interzone cells during the establishment of patellofemoral joint, immunostaining analysis of the knee samples showed that at E14.0, GDF5 was expressed in the interzone cells in Wt samples where GDF5 mostly co-expressed with SOX9-positive cells (Figure 3C). At E15.5, when the patellofemoral joint has been established in Wt samples, the expression of GDF5 was found in the vicinity of the patellofemoral joint where robust proliferating cells have been observed (Figures 3A–C). At both embryonic stages, the shape of the interzone cells was found flat and elongated (Figure 3C). On the contrary, *Rab23* ^*−/−*^ samples at E14.0–E15.5 expressed barely detectable GDF5, which failed to mark interzone cells at E14.0, and subsequently failed to develop a patellofemoral joint at E15.5 (Figure 3C).

### RAB23 regulates the co-expression of SOX9 and Scx in the fibrocartilaginous entheses

**FIGURE 4:**
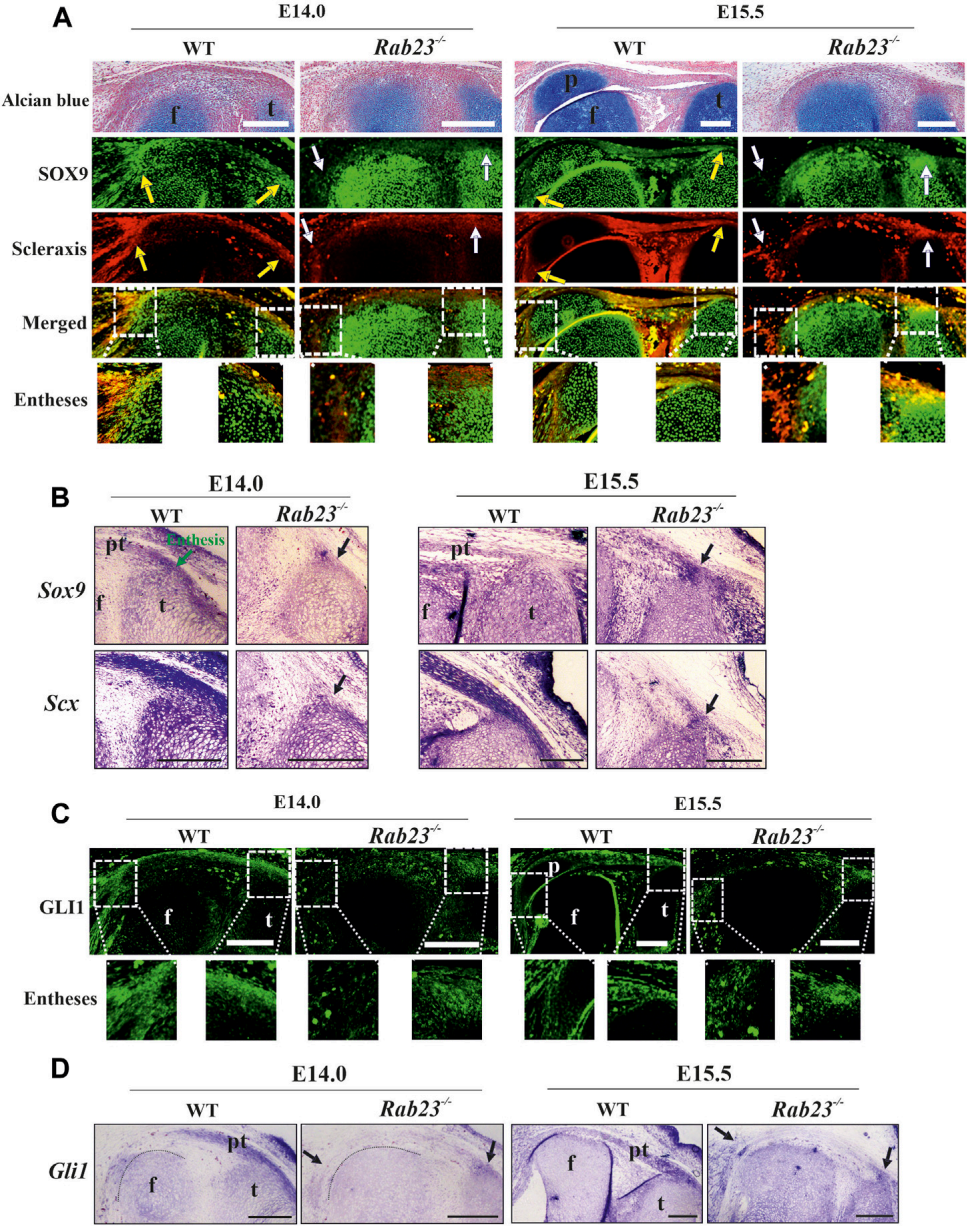
Scleraxis, SOX9, and GLI1 expression analysis during enthesis formation. **(A, B)** Immunohistochemical and *in situ* expression analysis of SOX9 and Scx in the Wt and *Rab23* ^*−/−*^ mice sagittal sections of the hind limbs obtained from E14.0 and E15.5 embryos. In Wt samples, both these markers are predominantly co-expressed in the junction between the quadriceps tendon and the patella (yellow arrow) **(A)** and also the patella tendon and the tibia (yellow and black arrows) at both the embryonic stages **(A, B)**. In *Rab23* ^*−/−*^ samples, since no patella was formed, co-expression of SOX9 and Scx is missing (white arrows) **(A)** except that the junction between the patella tendon-like structure and tibia gives rise to malformed entheses at E15.5 (white and black arrows) **(A, B)**. To highlight the cartilage cells of the patella, sagittal sections from E14.0 and E15.5 hind limbs are stained with Alcian blue and fast red (upper panel) (*n* = 3 samples for each age and genotype). p: patella, pt: patellar tendon, f: femur, and t: tibia. Scale bar: 200 μm. **(C, D)** Immunohistochemical and *in situ* expression analysis of Hh (Hedgehog) component Gli1 in the Wt and *Rab23* ^*−/−*^ mice sagittal-sectional tissues of hind limbs are obtained from E14.0 and E15.5 embryos. In Wt samples, Gli1 is predominantly expressed in the future patella, patella tendon, quadriceps tendon, rectus femoris, and vastus medialis tendon and in the meniscus at both the embryonic stages **(C, D)**. In *Rab23* ^*−/−*^ samples, Gli1 is weakly expressed throughout the E14.0 and E15.5 tissues and gives rise to the malformed entheses (black arrows) **(C, D)**. Black dotted marking indicates the end of the femur toward the patella **(D)** (*n* = 3 samples for each age and genotype). p: patella, pt: patellar tendon, f: femur, and t: tibia. Scale bar: 200 μm.

Our results show that RAB23 regulates the co-expression of SOX9 and Scx in the patella progenitors during their specification. SOX9 and Scx double-positive cells contribute to the establishment of a bridge between cartilages and tendons/ligaments, known as entheses (Sugimoto et al., 2013). A study showed GDF5-expressing cells giving rise to the fibrocartilaginous cells under the regulation of Hh signaling which later forms the enthesis (Dyment et al., 2015). To understand whether RAB23 regulates the co-expression of SOX9 and Scx in the entheses, immunohistochemical expression analysis of SOX9 and Scx in Wt and *Rab23* ^*−/−*^ samples at E14.0 and E15.5 revealed that Wt samples predominantly co-expressed SOX9 and Scx in the enthesis between the quadriceps tendon and the patella and also the enthesis that formed between the patella tendon and the tibia at both the embryonic stages (Figure 4A). In *Rab23* ^*−/−*^ samples, the co-expression of SOX9 and Scx was missing at both the embryonic stages except at the junction that formed between the patella tendon-like structure and tibia, which gave rise to malformed enthesis at E15.5 (Figure 4A). We have further examined the expression of *Sox9* and *Scx* in the fibrocartilaginous entheses by *in situ* hybridization. Our result reaffirmed the robust expression of *Sox9* and *Scx* in these structures in Wt samples at E14.0 and E15.5, whereas *Rab23* ^*−/−*^ samples showed a reduced expression of these markers at both embryonic stages (Figure 4B).

### Analysis of GLI1 expression in Wt and *Rab23* ^*−/−*^ entheses

We have previously reported that RAB23 regulates GLI1 during early calvarial bone and suture development (Hasan et al., 2020). Other studies on knee and synovial joint formation revealed that GLI1-positive cells contribute to the formation of entheses and ablation of Hh signaling in the tenocyte impairs matrix organization in the enthesis (Liu et al., 2012; Liu et al., 2013; Breidenbach et al., 2015; Schwartz et al., 2017). To understand whether RAB23 could show an effect on GLI1 expression during the fibrocartilaginous entheses formation around the knee, we analyzed GLI1 expression by IHC (Figure 4C) and by *in situ* hybridization (Figure 4D) in the Wt and *Rab23* ^*−/−*^ samples at E14.0 and E15.5, respectively. The result showed that GLI1 expression marked the fibrocartilaginous entheses that formed between the quadriceps tendon and patella and tibia and patella tendon in Wt samples. However, *Rab23* ^*−/−*^ samples developed only one enthesis between the patellar tendon-like structure and the tibia at both embryonic stages (Figures 4C, D). Analysis of GLI1 in the enthesis in *Rab23* ^*−/−*^ samples revealed that GLI1 expression was weak and diffuse (Figures 4C, D). Concomitant to these findings, we found that GLI1 showed poor expressions at the patellar tendon-like structure and in the fibrocartilaginous structure of the cruciate ligaments in *Rab23* ^*−/−*^ samples (Figures 4C, D).

### Aberrant expression of TGFβR2 and *phospho*-pErk44/42 in the *Rab23* ^*−/−*^ patellofemoral sample

TGFβ signaling has been shown to positively regulate *Scx* expression in early mouse limb explants and could direct mouse mesodermal stem cells toward the tendon lineage (Havis et al., 2014). TGFβ signaling is critical during patella bone initiation as the conditional knockout of *Tgfβr2* in mouse early limb mesenchymal cells cause failure of *Sox9* and *Scx* double-positive patella progenitor specification (Eyal et al., 2015). TGFβ signaling also has been implicated in committing mouse mesodermal stem cells toward the tendon lineage (Havis et al., 2014). However, in mice, the FGF/ERK MAPK pathway negatively regulates *Scx* expression in undifferentiated limb mesodermal cells (Havis et al., 2014). Our previous study showed that a deficiency of RAB23 caused aberrant FGF signaling (Hasan et al., 2020).

**FIGURE 5:**
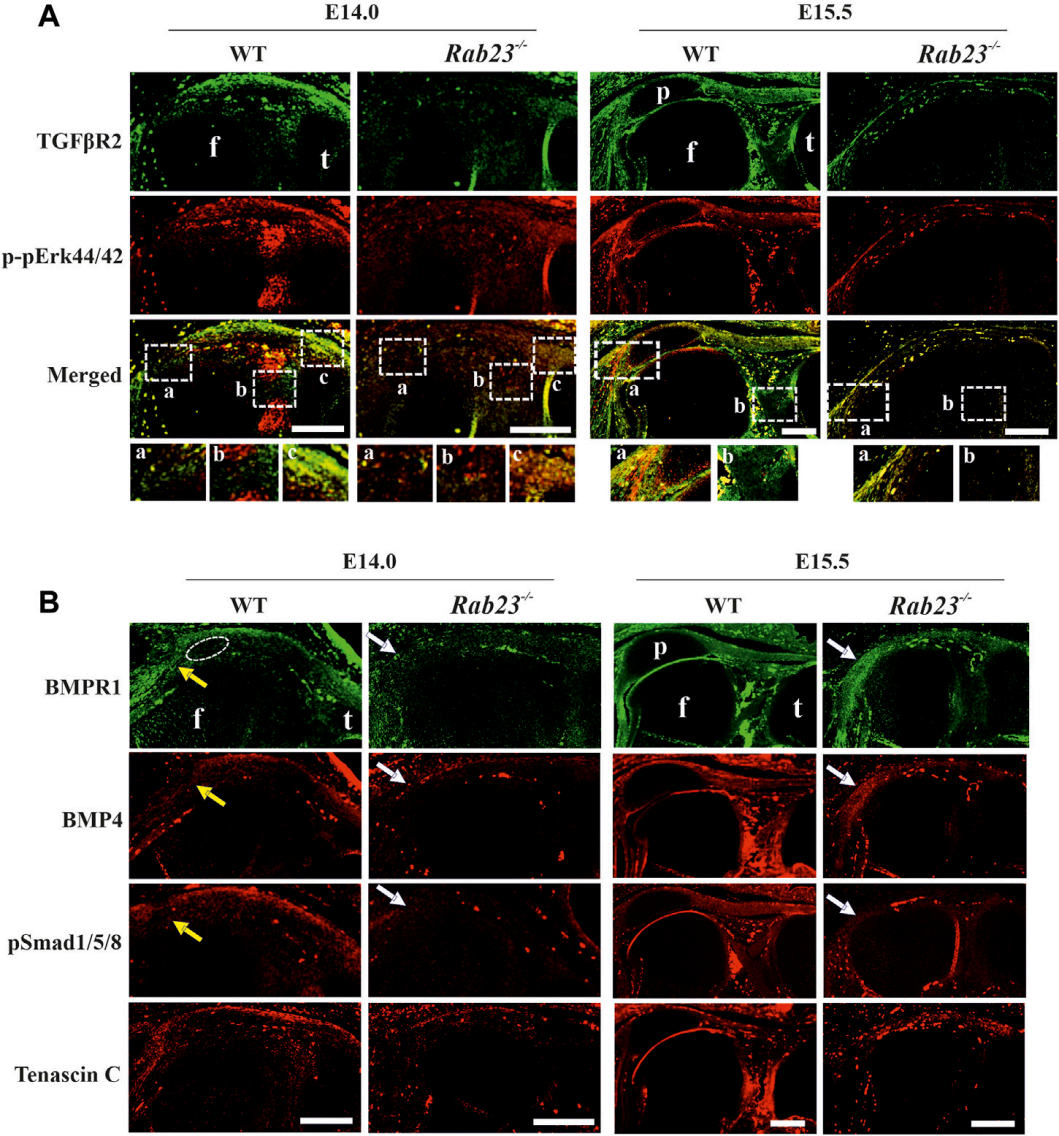
Analysis of TGFβR2, phospho-pErk44/42, and BMP signaling during patella, tendonogenesis, and ligamentogenesis. **(A)** Immunohistochemical expression analysis of TGFβR2 and phospho-pErk44/42 in sagittal sections of the hind limbs obtained from E14.0 and E15.5 Wt and *Rab23* ^*−/−*^ embryos. In Wt samples, TGFβR2 is expressed in the patella site, patella tendon, quadriceps tendon, rectus femoris, and vastus medialis tendon and also in the cruciate ligaments in Wt samples. In *Rab23* ^*−/−*^ samples, TGFβR2 expression remained rudimentary and aberrant at both the embryonic stages analyzed. The Wt sample at E14.0 shows the expression of phospho-pErk44/42 in the patellofemoral junction except for the cruciate ligament. At E15.5, phospho-pErk44/42 shows expression in the boundary cells of the patellofemoral structure and also beneath the quadriceps tendon. This expression was altered in *Rab23* ^*−/−*^ samples (*n* = 3 samples for each age and genotype). p: patella, f: femur, and t: tibia. Scale bar: 200 μm. **(B)** Immunohistochemical expression analysis of BMP signaling components BMP4, BMPR1, and pSMAD 1/5/8 in sagittal sections of the hind limbs obtained from E14.0 and E15.5 Wt and *Rab23* ^*−/−*^ embryos. BMPR1, BMP4, and pSMAD 1/5/8 expression images show that all these markers are expressed in the patella site, patella tendon, quadriceps tendon, rectus femoris, and vastus medialis tendon (yellow arrow) in E14.0 and E15.5 samples. In *Rab23* ^*−/−*^ samples, BMPR1, BMP4, and pSMAD 1/5/8 expressions are barely detectable in these structures at E14.0 (white arrow) and show expression mostly in the femur head toward the rectus femoris and vastus medialis tendon (white arrow) at E15.5. To highlight the structural protein in the tendon and ligament cells, sagittal sections from E14.0 and E15.5 hind limbs were stained with Tenascin-C (lower panel). Dotted circle indicates the future patella site. (*n* = 3 samples for each age and genotype). p: patella, f: femur, and t: tibia. Scale bar: 200 μm.

Here, we showed that *Rab23* ^*−/−*^ mice failed to specify patella progenitors and largely affected the Scx expression. We then try to understand whether RAB23 deficiency could affect the expression of TGFβR2 and phospho-pErk44/42. We analyzed TGFβR2 and phospho-pErk1/2 (44/42) expression in Wt and *Rab23* ^*−/−*^ patellofemoral samples at E14.0 and E15.5, respectively. We found that TGFβR2 was expressed in the future patella site, in the patella tendon, quadriceps tendon, entheses, and in the cruciate ligaments in Wt samples at E14.0 and E15.5 (Figure 5A). In contrast, *Rab23* ^*−/−*^ patellofemoral samples showed aberrant and rudimentary TGFβR2 expression in these embryonic stages (Figure 5A). Analysis of phospho-pErk44/42 in Wt samples showed its expression in the patella tendon and most prominently in the patellofemoral junction except cruciate ligaments, and the expression was also observed around the edge of the femur head except that the patella forming site at E14.0 (Figure 5A). At E15.5, the phospho-pErk44/42 expression persisted in the boundary cells of patellofemoral structure, and the expression was also observed beneath the quadriceps tendon (Figure 5A). *Rab23* ^*−/−*^ patellofemoral samples showed aberrant and reduced expression of phospho-pErk44/42 in both embryonic stages (Figure 5A).

### Aberrant BMP4/BMPR1/pSmad1/5/8 expression during patella formation in *Rab23* ^*−/−*^ mice

As BMP signaling is known to regulate patella formation (Eyal et al., 2015; Eyal et al., 2019), we analyzed BMP4, BMPR1, and pSmad1/5/8 expression in Wt and *Rab23* ^*−/−*^ samples at E14.0 and E15.5. In *Rab23* ^*−/−*^ samples, we found a loss of expression of BMPR1, BMP4, and pSmad1/5/8 in the patella progenitor cells (Figure 5B). Also, these proteins were barely expressed in the patellar tendon-like structure and in the quadriceps tendon-like structures in *Rab23* ^*−/−*^ samples (Figure 5B).

### RAB23 deficiency causes aberrant patella tendon and cruciate ligament formation

**FIGURE 6:**
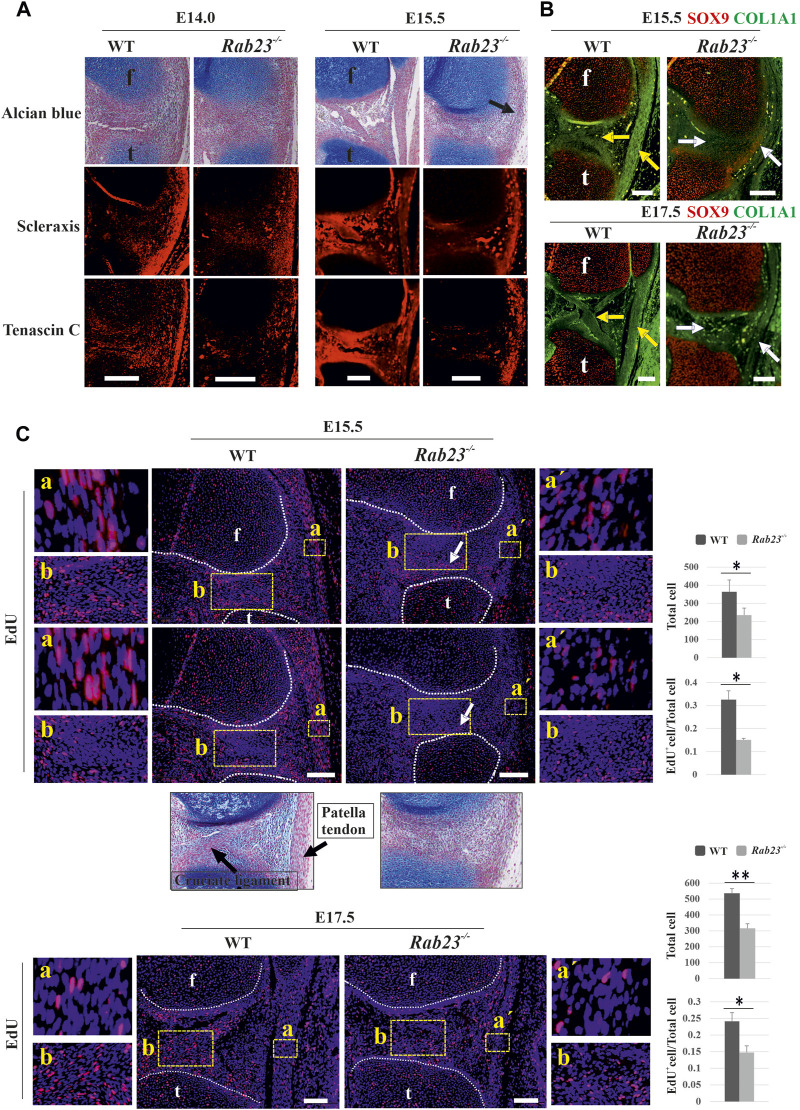
RAB23 regulates tendonogenesis and ligamentogenesis and cell proliferation in the patella tendon and cruciate ligaments. **(A)** Immunohistochemical expression analysis of early tenocyte-expressing marker Scx and structural protein marker Tenascin-C in the Wt and *Rab23* ^*−/−*^ mice in the sagittal sections of hind limbs obtained from E14.0 and E15.5. Wt samples show Scx and Tenascin-C expression in the patella tendon at both the embryonic stages, where Tenascin-C confirms well-developed patella tendon. *Rab23* ^*−/−*^ samples at E14.0 shows less Scx expression in the patella tendon-like structure, and as *Rab23* ^*−/−*^ samples at E15.5 failed to develop patella and patella tendon, a musculostructure connected the femur to the tibia (arrow). Scx is expressed in this structure; however, subsequent Tenascin-C expression revealed that musculostructures are disrupted in *Rab23* ^*−/−*^ samples. Similar to patella tendon, analysis of Scx and Tenascin-C expressions in the cruciate ligaments show that in Wt samples, both markers are expressed well to mark the cruciate ligaments. However, both markers failed to mark cruciate ligaments in *Rab23* ^*−/−*^ samples. To highlight the cartilage cells of the patella, patella tendon sagittal sections from E14.0 and E15.5 hind limbs were stained with Alcian blue and fast red (upper panel) (*n* = 3 samples for each age and genotype). f: femur and t: tibia. Scale bar: 200 μm. **(B)** Immunohistochemical expression analysis of SOX9 and structural integrity protein of tendon and ligaments COL1A1 in the Wt and *Rab23* ^*−/−*^ mice sagittal-sectional tissues of hind limbs obtained from E15.5 and E17.5. Wt samples show COL1A1 expression that marks the cruciate ligament and patella tendon at both the embryonic stages (yellow arrow). However, *Rab23* ^*−/−*^ samples show COL1A1 expression that barely detected cruciate ligament at E15.5 (white arrow) and failed to demonstrate the cruciate ligaments at E17.5 (white arrow). *Rab23* ^*−/−*^ samples failed to develop patella and showed weaker COL1A1 expression in the ligament-like structure that connects the femur and the tibia. (*n* = 3 samples for each age and genotype). f: femur and t: tibia. Scale bar: 200 μm. **(C)** EdU pulsed cell proliferation analysis of patella tendon **(A)** and patella tendon-like structure (a´) in Wt and *Rab23* ^*−/−*^ samples at E15.5 and E17.5. Wt samples at both the embryonic stages show significantly more cell proliferation in the patella tendon **(A)** compared to patella tendon-like (a´) structure in *Rab23* ^*−/−*^ samples as the counting of all cells (blue + EdU + cells) and the ratio of EdU^*+*^ cells (EdU^*+*^ cells/all cells) are higher in Wt samples. *Rab23* ^*−/−*^ patella tendon-like structure is malformed at both E15.5 and E17.5 samples. Further observations by Alcian blue and fast red staining revealed that *Rab23* ^*−/−*^ samples fail to develop cruciate ligaments, therefore, EdU-positive cells in *Rab23* ^*−/−*^ samples show an irregular pattern of cell proliferation compared to Wt samples in the junction between tibia and femur **(B)** (white arrow). (*n* = 3 samples for each age and genotype). Nuclei were stained with Hoechst (blue), EdU^*+*^ cells (red). Statistical significance was defined as *p* value ˂ 0.05 (*) and *p* value ˂ 0.02 (**). f: femur and t: tibia. Scale bar: 100 μm.

To understand whether RAB23 shows the regulation of tenocyte in the development of the patella tendon and cruciate ligament, we analyzed the expression of Scx, an early marker for tendon and ligament progenitors, and Tenascin-C, a structural matrix protein marker for tendon and ligament in Wt and *Rab23* ^*−/−*^ samples at E14.0 and 15.5. The results showed that *Rab23* ^*−/−*^ samples represented reduced and disorganized scleraxis expression in the cruciate ligament (Figure 6A). During the analysis of the structural protein Tenascin-C, a downstream target gene of Scx revealed that Tenascin-C was poorly expressed in the cruciate ligament and in the meniscus in *Rab23* ^*−/−*^ samples (Figure 6A). The patella tendon, which connected the patella to the tibia, showed robust expression of Scx in Wt samples. In *Rab23* ^*−/−*^ samples, as the patella was not formed, the patella tendon also failed to develop; instead, a ligament-like structure connected the femur to the tibia (Figure 6A). Scx and Tenascin-C expression in this structure was found aberrant and disorganized. We further aimed to follow the development of this structure including the cruciate ligament. Here, we analyzed the expression of COL1A1, another matrix protein of tenocytes, which provides structural integrity to the cruciate ligament and the patella tendon. The results show that *Rab23* ^*−/−*^ mice at E15.5 and E17.5 failed to mark the cruciate ligament by COL1A1 and the patella tendon-like structure was significantly thin and malformed. The Wt samples showed robust expression of COL1A1 and marked very well the patella tendon and cruciate ligament (Figure 6B). These results collectively indicate that RAB23 regulates tendonogenesis and ligamentogenesis of the knee.

We have previously reported that RAB23 regulates cell proliferation during early calvarial bone and suture development (Hasan et al., 2020). To understand whether RAB23 could influence cell proliferation during patella tendon and ligament development, we analyzed EdU pulsed Wt and *Rab23* ^*−/−*^ patella tendon samples at E15.5 and E17.5 (Figure 6C). The patella tendon in Wt samples showed robust cell proliferation in E15.5 and E17.5. However, *Rab23* ^*−/−*^ samples developed a tendon-like structure instead of a patella tendon which showed significantly less proliferative cells at both the embryonic stages (Figure 6C). Further observations of the region of cruciate ligaments in *Rab23* ^*−/−*^ samples showed the pattern of proliferation in this region was aberrant than Wt samples (Figure 6C). While Wt samples showed proliferating cells in and around the cruciate ligaments, *Rab23* ^*−/−*^ samples showed less proliferative cells in the region of cruciate ligaments; instead, *Rab23* ^*−/−*^ samples showed proliferative cells closer to the articular cartilage site of the tibia (Figure 6C). Moreover, we showed that the cruciate ligaments in *Rab23* ^*−/−*^ samples were poorly detectable at E15.5 samples (Figure 6C).

### *Rab23* ^*−/−*^ mice show multiple skeletal patterning defects

**FIGURE 7:**
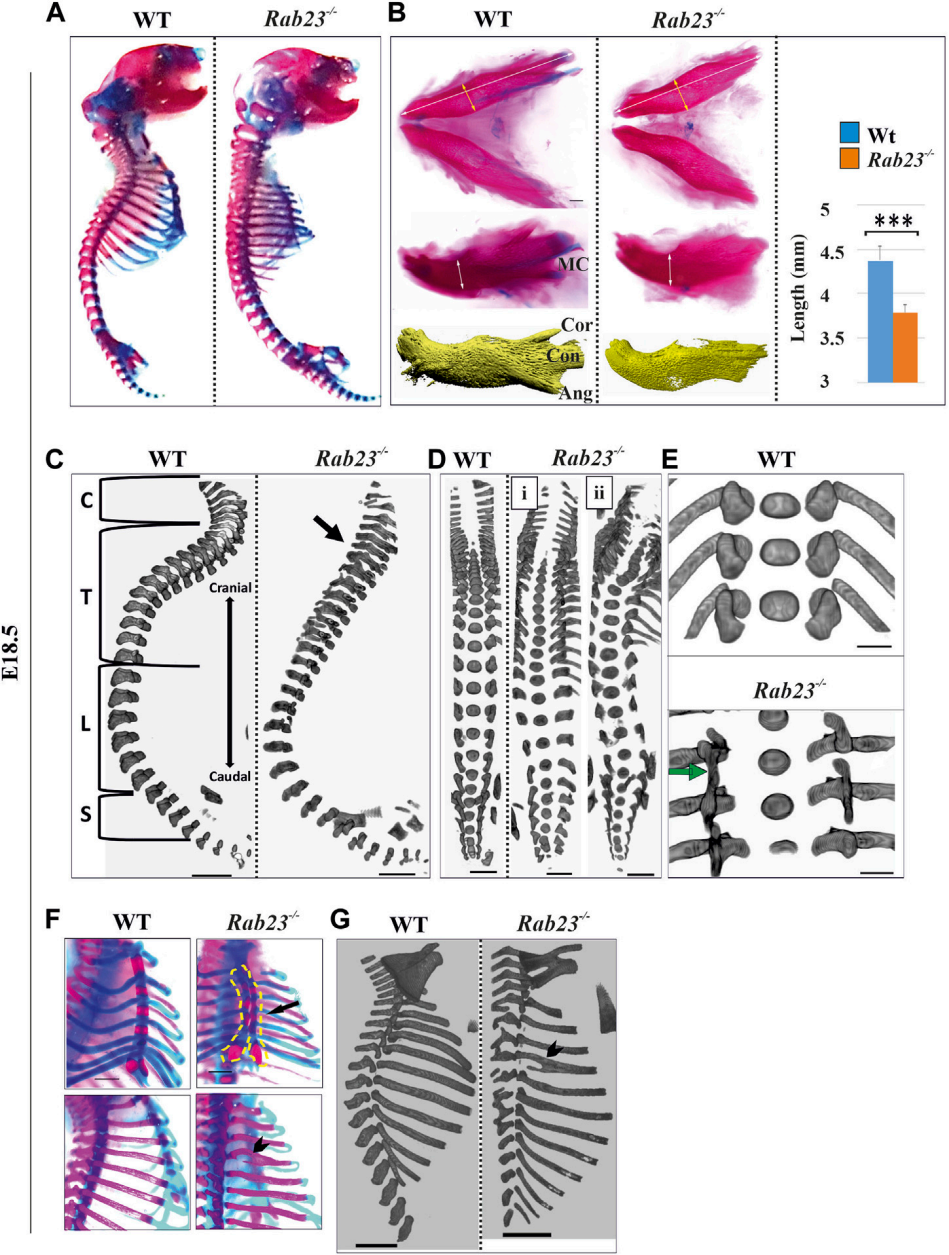
*Rab23* ^*−/−*^ mice show diverse skeletal phenotypes. **(A)** Alizarin red (bone) and Alcian blue (cartilage) staining of whole WT and *Rab23* ^*−/−*^ mice at E18.5 showing the craniofacial, axial, and appendicular skeleton except for the fore and hind limbs (*n* = 5 for each age and genotype). **(B)** Alizarin red, Alcian blue staining, and µ-CT images of the mandible of Wt and *Rab23* ^*−/−*^ mice at E18.5. The mandibular processes; cor: coronoid process, con: condylar process, and ang: angular processes are poorly developed in *Rab23* ^*−/−*^ mice. *Rab23* ^*−/−*^ mandibles are smaller than Wt samples (*n* = 6 for each age and genotype). Statistical significance was defined as *p* value ˂ 0.001 (***). **(C–E)** μ-CT images of the vertebral column of Wt and *Rab23* ^*−/−*^ mice at E18.5. *Rab23* ^*−/−*^ mice show neck flexure defect (C. arrow), scoliosis (D), and fused vertebrae thoracic T6-T8 (E, arrow). Scale bar: 1 mm (C, D). **(F, G)** Alizarin red, Alcian blue staining, and µ-CT images of ribs of Wt and *Rab23* ^*−/−*^ mice at E18.5. *Rab23* ^*−/−*^ mice show split sternum (F, arrow) and bifid ribs (F and G, arrowhead) (*n* = 5 for each age and genotype). Scale bar: 1.5 mm **(E)**, 1 mm **(F)**, and 2 mm **(G)**.

We further analyzed the skeleton of *Rab23* ^*−/−*^ mice and compared them with Wt littermates to understand the global effect of RAB23 deficiency on skeletal development and patterning. Here, we found that *Rab23* ^*−/−*^ mice show multiple axial and appendicular skeletal patterning defects (Figure 7). Our results demonstrated that *Rab23* ^*−/−*^ mice show the craniofacial bones, including parietal, occipital, and palatine bones, are misshaped (Table 1) and showed shorter mandibular bones including malformed coronoid, condylar, and angular processes (Figure 7B). The Meckel’s cartilage in *Rab23* ^*−/−*^ mice are largely missing (Figure 7B). Analysis of the vertebral column showed neck curvature defect, scoliosis (Figures 7A–C), fused vertebral column, defective curvature of the ribs, fused ribs, bifid ribs, and bifurcated sternum (Figures 7D–G). In addition to that, *Rab23* ^*−/−*^ mice showed malformed deltoid tuberosity (Supplementary Figure S5) and defective joint morphogenesis (Supplementary Figure S6).

**TABLE 1:** Skeletal phenotypic resemblance between *Rab23* ^*−/−*^ and *Tgfβ2* ^*−/−*^ mice.

Skeletal phenotypes	Shown in *Rab23* ^*−/−*^ mice	Shown in *Tgfβ2* ^*−/−*^ mice	
Cranial bone defects			
*Frontal bone*	*Yes*	*Yes*	
*Parietal bone*	*Yes*	*Yes*	
*Interparietal bone*	*Yes*	*Yes*	
*Occipital bone*	*Yes*	*Yes*	
*Squamous bone*	*Yes*	*Yes*	
*Palatine bone*	*Yes*	*Yes*	
Anterior frontanelle defect			
*Wider*	*Yes*	*Yes*	
Mandibular bone defects			
*Shorter mandible*	*Yes*	*Yes*	
*Coronoid process*	*Yes*	*Yes*	
*Condylar process*	*Yes*	*Yes*	
*Meckel’s cartilage*	*Yes*	*Yes*	
Long bone defects			
*Shorter*	*Yes*	*Yes*	
*Radius and ulna*	*Yes*	*Yes*	
*Deltoid tuberosity*	*Yes*	*Yes*	
Spinal and rib defects			
*Kyphoscoliosis*	*Yes*	*Yes*	
*Fused column*	*Yes*	*Yes*	
*Curvature*	*Yes*	*Yes*	
*Fused ribs*	*Yes*	*Yes*	
*Wavy irregular ribs*			
*Bifid ribs*	*Yes*	*Yes*	
Sternum defect			
*Bifurcation*	*Yes*	*Yes*	
Other related phenotypes	***Rab23*** ^***−/−***^ **mice**	**Disrupted Tgfβ2 signaling**	
Patella			
*Missing*	*Yes*	*Yes*	
Knee tendon and ligament			
*Missing*	*Yes*	*Yes*	
Joint morphogenesis			
*Defective*	*Yes*	*Yes*	

## Discussion

In this study, we demonstrate that the small GTPase protein RAB23 plays a critical role in knee and knee joint development. The knee bone, also known as the patella, functions during movement: providing structural support and stability to the knee joint (Lennox et al., 1994; Sarin et al., 1999). Patella is the largest sesamoid bone in mammals known to originate from Scx and SOX9 double-positive patella chondroprogenitor cells (Eyal et al., 2015; Eyal et al., 2019). We show that RAB23-deficient mice fail to develop patella and that missing patella in *Rab23* ^*−/−*^ mice was not due to the programmed cell death (Supplementary Figure S1). We demonstrate that apart from SOX9 expression, RAB23 deficiency broadly misregulated Scx expression, which is essential for patella progenitor, patella tendon, quadriceps tendon, cruciate ligament formation, and their entheses formation and at the same time establishment of the patellofemoral joint. Our study revealed that SOX9 and Scx double-positive patella chondroprogenitor populations were lost in the patella site in *Rab23* ^*−/−*^ mice (Figure 2). Since no patella anlagen formed in *Rab23* ^*−/−*^ samples, the expansion of chondrogenesis has been observed toward the femur head (Figure 3 and Supplementary Figure S4). The missing patella also has been noticed in mice with conditional inactivation of *Sox9* (Sugimoto et al., 2013)*.*

After the initiation of patella anlagen, the mechanical load is required for patellofemoral joint formation. The autosomal recessive mutation in *mdg* (muscular dysgenesis) mice, which lack contractility of skeletal muscles, results in a failure of patellofemoral joint development with the patella remains in part of the femur (Pai, 1965a; Pai, 1965b; Eyal et al., 2015). We observed that while collecting the embryos at E18.5, Wt embryos could stretch their hind limbs, however, *Rab23* ^*−/−*^ mice hind limbs remained static. Similarities between mdg mutant mice and *Rab23* ^*−/−*^ mice may suggest that lack of skeletal muscle contraction could contribute to the patellofemoral phenotypes observed in *Rab23* ^*−/−*^ mice. Defective joint formation also has been reported in TGFβ2 deficient mice (Spagnoli et al., 2007; Wang et al., 2014; MacFarlane et al., 2017). Patellofemoral joint establishment also requires higher cell proliferation in the boundary cells and in the interzone cells, where the cells gradually lose their chondrocyte characteristics and become flat and elongated (Eyal et al., 2015). Such events were observed missing in *Rab23* ^*−/−*^ samples. Also, *Rab23* ^*−/−*^ samples failed to organize GDF5-expressing boundary cells (Figure 3). Scx is known as the earliest marker for tendon and ligament development (Schweitzer et al., 2001; Kuo and Tuan, 2008). We found that Scx, along with matrix structural proteins COL1A1 and Tenascin-C, is largely affected in *Rab23* ^*−/−*^ mice which rendered defective tendonogenesis, ligamentogenesis, and joint formation (Figure 6 and Supplementary Figure S3). FGF/ERK MAPK signaling, which is implicated as a positive regulator of *SCX* expression in chick undifferentiated limb cells, functions negatively on *Scx* expression in mouse undifferentiated limb mesodermal cells (Havis et al., 2014; Havis et al., 2016). In mouse patellofemoral samples, we find that RAB23 deficiency affected FGF/ERK expression (Figure 5). Our previous study also showed that RAB23 deficiency affects FGF/ERK signaling during early mouse calvarial bone and suture development (Hasan et al., 2020). A study showed that ectodermal signals cause the induction of tendon progenitors in the lateral plate mesoderm (LPM), and subsequently, LPM gives rise to the limb tendons which are Scx positive (Schweitzer et al., 2001). Upon induction, TGFβ signaling is required for the maintenance of tendon progenitors, and thus, *Tgfβ2* ^*−/−*^ *; Tgfβ2* ^*−/−*^ mice failed to develop limb tendons (Pryce et al., 2009). Here, we suggest that similar to TGFβ signaling, RAB23 is required for the maintenance of tendon progenitors.

The TGFβ2 signaling pathway plays a pivotal role during early fetal and embryonic development in mice (Sanford et al., 1997; Dunker and Krieglstein, 2002; Oka et al., 2007). Our study showed that TGFβR2 is expressed in the future patella site and in all the tendon and ligaments in Wt patellofemoral samples, and in *Rab23* ^*−/−*^ samples, TGFβR2 expression was rudimentary and aberrant (Figure 5). This phenomenon suggests that RAB23 might regulate Scx expression through TGFβ2 signaling. BMP signaling is required for the growth and differentiation of patella progenitors to form patella anlagen, and subsequently, patella anlagen separates from the femur by mechanical stimuli from patella tendon and quadriceps tendon and incorporates into the patella tendon by establishing the patellofemoral joint. Patellofemoral joint formation requires the expression of genes *Gdf5* and *Tnmd* that marks the interzone (Eyal et al., 2015; Eyal et al., 2019). Hedgehog signaling has been shown to be essential for limb patterning and growth plate chondrocyte proliferation and differentiation (Long et al., 2001). GLI1, which is the readout of Hh signaling, is expressed in the fibrocartilage junction, also known as enthesis (Liu et al., 2012) and is required for the differentiation, establishment, and maturation of entheses (Liu et al., 2013; Felsenthal et al., 2018; Zhang et al., 2020). A study showed that tendon-bone attachment cells are bi-fated and activates a mixture of tenocyte and chondrocyte transcriptomes (Kult et al., 2021). The establishment of such entheses requires Scx and SOX9 double-positive progenitors (Sugimoto et al., 2013), and the function of Scx is further extended as it is also required for the maturation of the entheses (Yoshimoto et al., 2017). The disruption of GLI1, Scx, and SOX9 expressions in *Rab23* ^*−/−*^ mice could explain why the musculoskeletal system was defective in these mice.

Our results suggest that RAB23 is required for the specification of patella chondroprogenitor cells, which co-expressed SOX9 and scleraxis (Figure 2). Since RAB23 is necessary for TGFβR2 and scleraxis expression during early embryonic stages, it, therefore, contributes to tendonogenesis and ligamentogenesis (Figures 5, 6). RAB23 modulation of GLI1, SOX9, and scleraxis expression collectively showed a role of RAB23 in the formation of entheses, also known as the musculoskeletal bridge (Figure 4). Meanwhile, BMP signaling is required for the subsequent growth and development of the patella anlagen to patella, and here, we showed that RAB23 deficiency largely affected the expression of BMP signaling components (Figure 5). Interestingly, a study on mice showed that limb-specific ablation of BMP2 can initiate patella bone formation but resulted in patella bone hypoplasia (Eyal et al., 2019). However, limb-specific ablation of BMP4 mice resulted in patella aplasia (Eyal et al., 2015; Eyal et al., 2019). These findings collectively indicate that BMP2 is required for the growth of patella bone and BMP4 is required for patella progenitor differentiation (Eyal et al., 2019). Since BMP2 and BMP4 may play downstream roles in TGFβ2 signaling during patella formation, we suggest that RAB23 may regulate BMP signaling either directly or indirectly *via* TGFβ signaling. The rationale behind the latter hypothesis is that RAB23, as a small GTPase of membrane trafficking protein, might be involved in TGFβR2 regulation by cargo turnover, relaying signals, receptor recycling, or degradation. Such function of RAB23 in the regulation of TGFβ2/BMP signaling might not be limited to just patella development but also the broader spectrum of musculoskeletal development. Since RAB23 and TGFβ2 signaling both regulate Scx expression to give rise to the patella progenitor cells and subsequent common steps in the development of the patella and surrounding tendons and ligaments, we compared the wider skeletal patterning phenotypes of *Tgfβ2* ^*−/−*^ and *Rab23* ^*−/−*^ mice (Sanford et al., 1997). They showed great similarities. *Tgfβ2* ^*−/−*^ mice exhibit multiple axial and appendicular skeletal patterning defects including kyphoscoliosis, fused vertebral column, abnormal curvature of the ribs, fused ribs, bifid ribs, and bifurcation of the sternum in mice (Sanford et al., 1997; Oka et al., 2007). *Rab23* ^*−/−*^ mice replicate all these specific skeletal phenotypes (Figure 7; Table 1) including malformed deltoid tuberosity (Supplementary Figure S5) and defective joint morphogenesis (Supplementary Figure S6). Our findings and phenotypic similarities between *Tgfβ2* ^*−/−*^ and *Rab23* ^*−/−*^ mice highlight the possible cross-talk between RAB23 and TGFB2 signaling during musculoskeletal development and patterning.

## Data Availability

The original contributions presented in the study are included in the article/Supplementary Material. Further inquiries can be directed to the corresponding author.

## Supplementary material

Supplementary Figure S1 Apoptosis analysis in the developing patella in Wt and *Rab23* ^*−/−*^ mice. Tunnel staining was performed to show the apoptotic cells in the future patella site in Wt and *Rab23* ^*−/−*^ mice at E14.0 and E15.5. Positive control (DNase I treated) that shows the effectiveness of the tunnel kit and E15.5 *Rab23* ^*−/−*^ femur sample showed apoptotic cells (arrows) in the hypertrophic chondrocytes. (n = 3 for each age and genotype). f: femur, t: tibia, and pt: patellar tendon. Scale bar: 200 µm.

Supplementary Figure S2 Validation of RAB23 antibody. Validation of RAB23 antibody on the sagittal sections of hind limbs in *Rab23* ^*−/−*^ mice at E14.0 and E15.5. Alexa fluor 488 (green) was used to detect RAB23 primary antibody. Blue indicates nuclear staining. f: femur and t: tibia. Scale bar: 200 µm.

Supplementary Figure S3 COL1A1 expression analysis in Wt and *Rab23* ^*−/−*^ quadriceps tendon. Immunohistochemical expression of SOX9 and structural integrity protein of tendon and ligaments COL1A1 in the Wt and *Rab23* ^*−/−*^ mice in sagittal sections of hind limbs at E15.5 and E17.5. Wt samples show COL1A1 expression that demonstrates the quadriceps tendon including the rectus femoris tendon at both the embryonic stages (yellow arrow). However, *Rab23* ^*−/−*^ samples show COL1A1 expression that barely detects the quadriceps tendon including the rectus femoris tendon at both the embryonic stages (white arrow). (n = 3 for each age and genotype). f: femur and t: tibia. Scale bar: 200 µm.

Supplementary Figure S4 COL2A1 expression analysis in Wt and *Rab23* ^*−/−*^ patellofemoral samples. Image shows the staining of the chondrocyte marker COL2A1 in Wt and *Rab23* ^*−/−*^ samples at E15.5 and E17.5. COL2A1 shows more prominent expression in the articular cartilage toward the femur and tibia heads in *Rab23* ^*−/−*^ samples (white arrow), while the Wt samples exhibit a more uniform expression of COL2A1 throughout the femur and tibia heads. (n = 3 for each age and genotype). ac: articular cartilage, f: femur, t: tibia, and p: patella. Scale bar: 200 µm.

Supplementary Figure S5
*Rab23* ^*−/−*^ mice show malformed deltoid tuberosity in the forelimbs Alizarin red (bone) and Alcian blue (cartilage) staining of WT and *Rab23* ^*−/−*^ mice at E18.5. Forelimbs of *Rab23* ^*−/−*^ mouse has a poorly formed deltoid tuberosity in the humerus (arrow). h: humerus, r: radio, u: ulna, and dt: deltoid tuberosity. (n = 4 for each age and genotype).

Supplementary Figure S6 Abnormal joints in *Rab23* ^*−/−*^ hind limbs. Whole mount Alcian blue and alizarin red staining of Wt and *Rab23* ^*−/−*^ mice hind limbs at E18.5. The rudimentary tibiofemoral joint is abnormal in *Rab23* ^*−/−*^ mice (black arrows). In *Rab23* ^*−/−*^ mice, the fibula and tibia bones are attached to the head of the femur (black arrows, insets, upper and lower panels). The joint between the tarsal and the metatarsal is also morphologically abnormal with a resultant flex of the foot in *Rab23* ^*−/−*^ mice (insets, lower and upper panels, white arrows). (n = 4 for each age and genotype).
