# Supplementary figures and images for: RAB23 regulates musculoskeletal development and patterning
Source: Front Cell Dev Biol. 2023 Feb 23;11:1049131. doi: 10.3389/fcell.2023.1049131 (PMC9995984; doi:10.3389/fcell.2023.1049131)

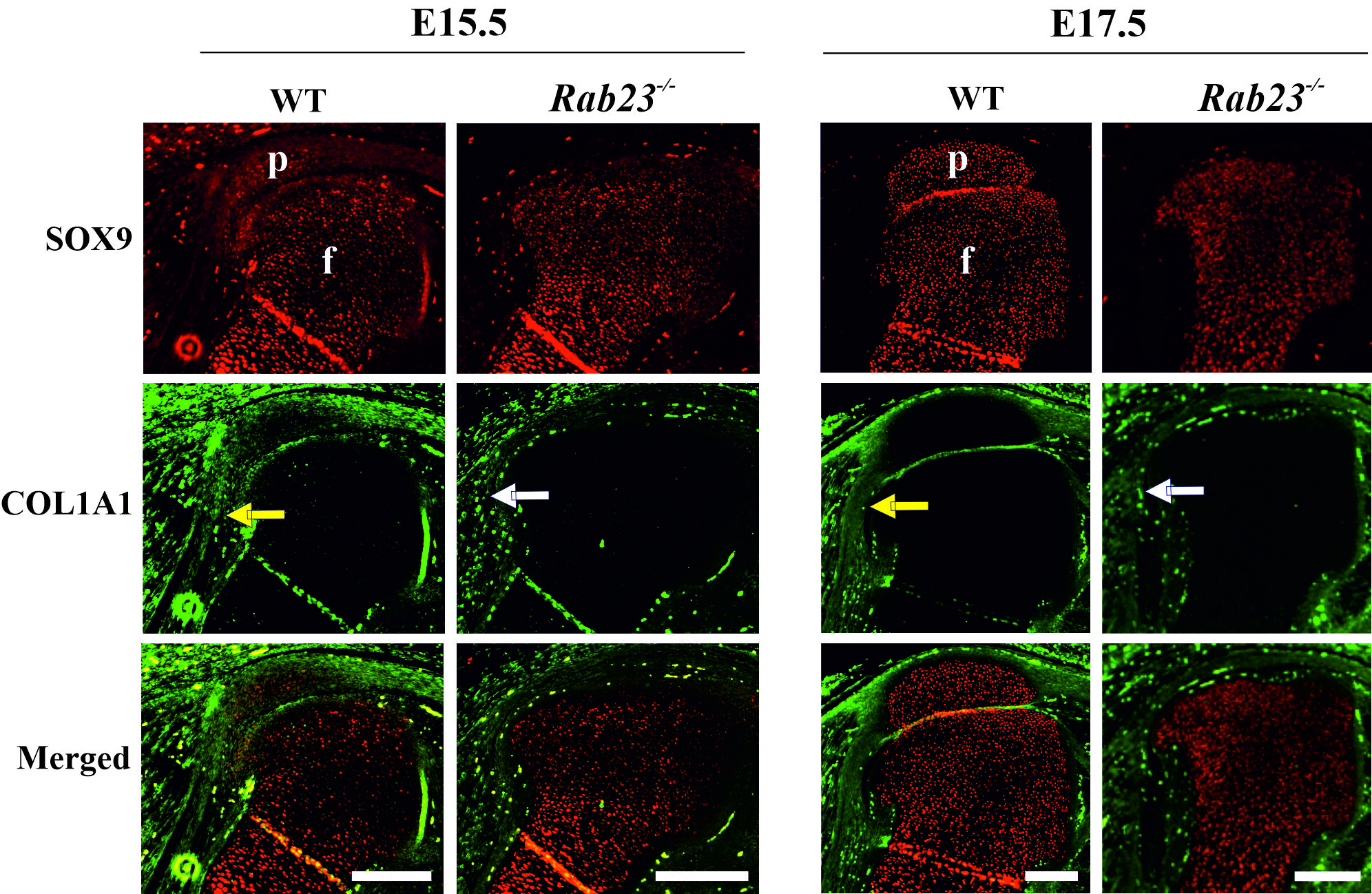

Supplement: Supplementary file 1 [file Image3.JPEG]

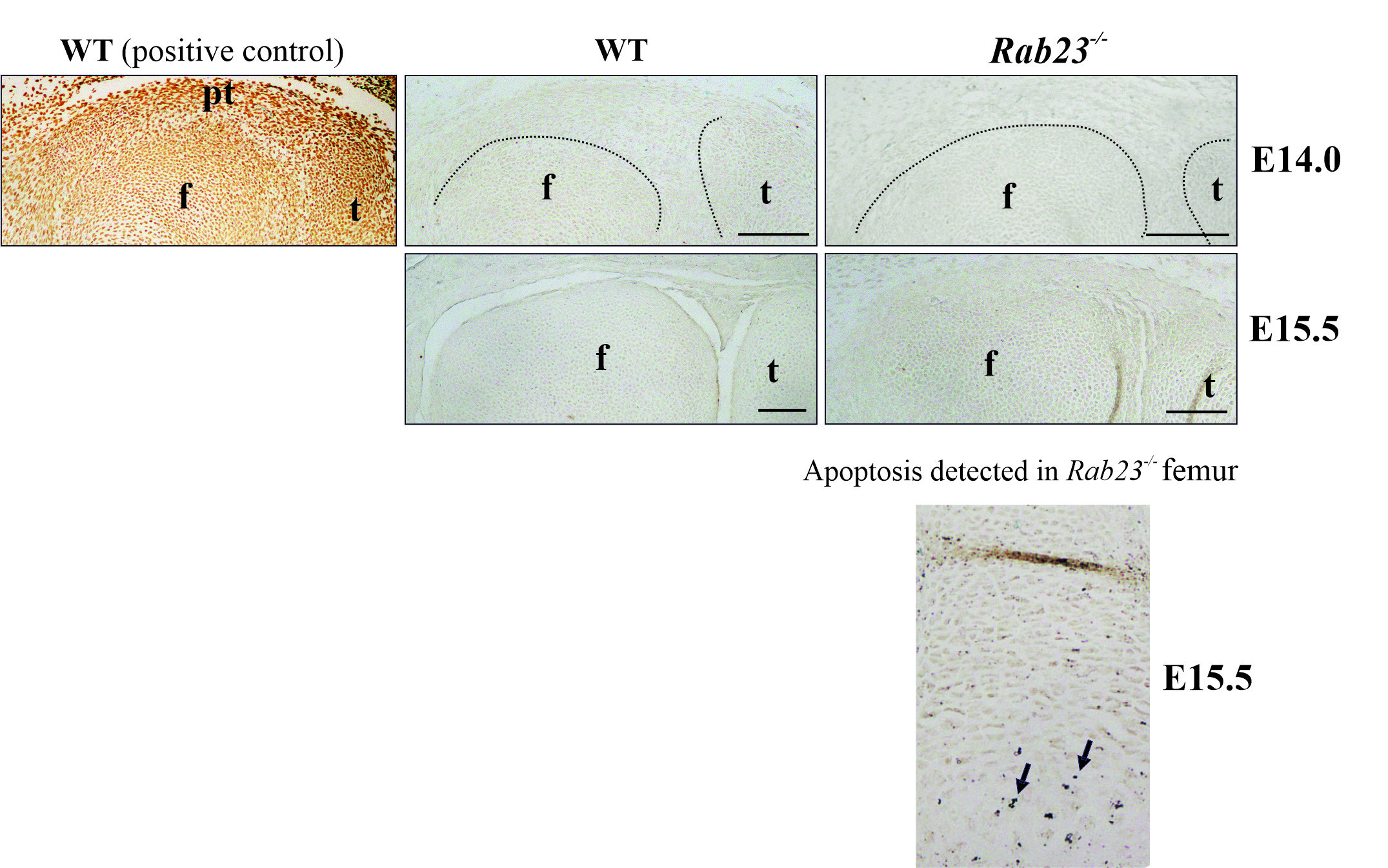

Supplement: Supplementary file 2 [file Image1.JPEG]

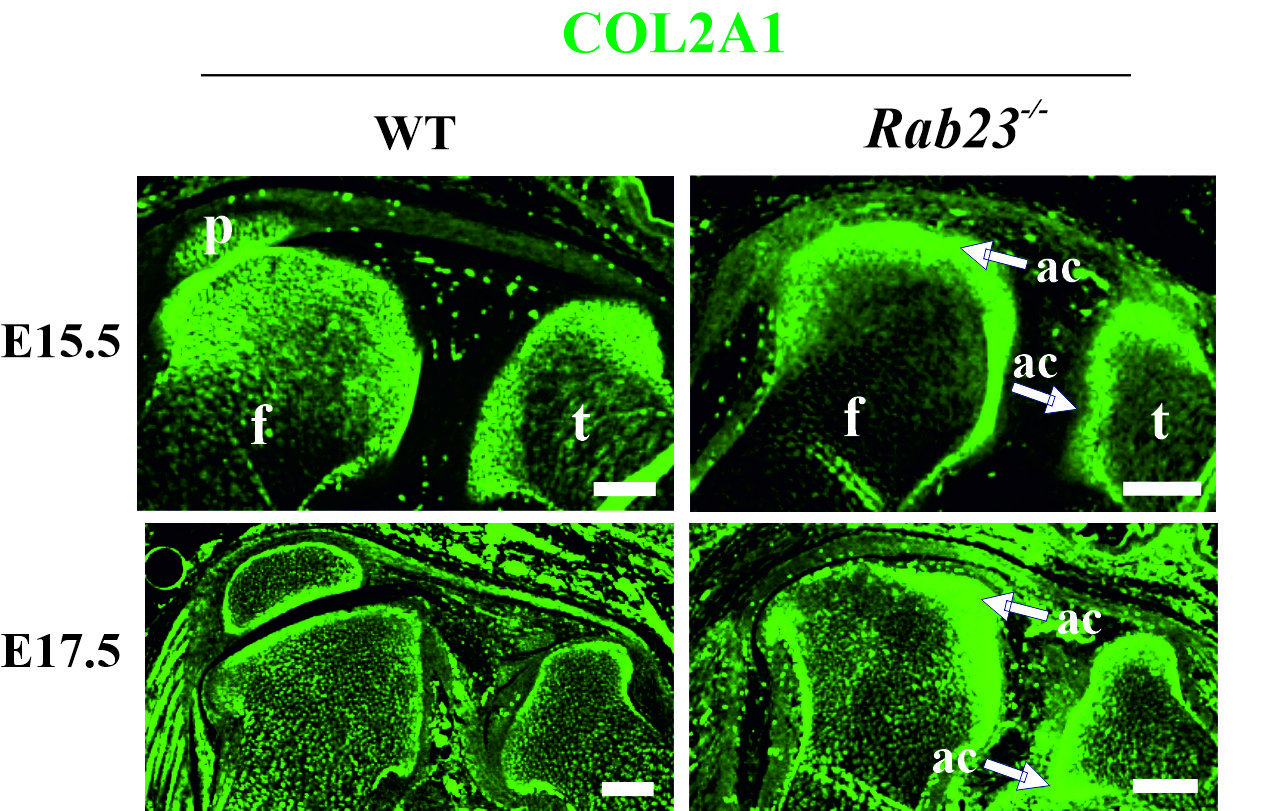

Supplement: Supplementary file 3 [file Image4.JPEG]

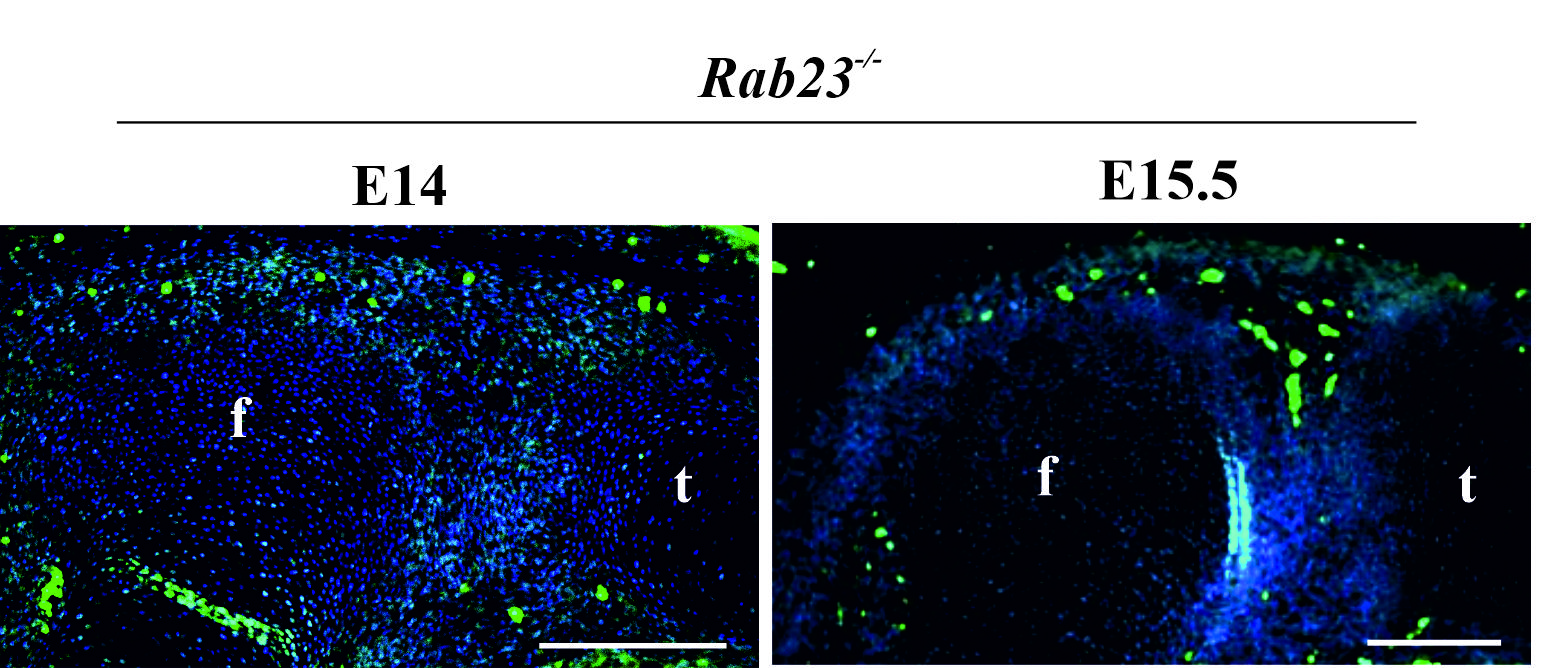

Supplement: Supplementary file 4 [file Image2.JPEG]

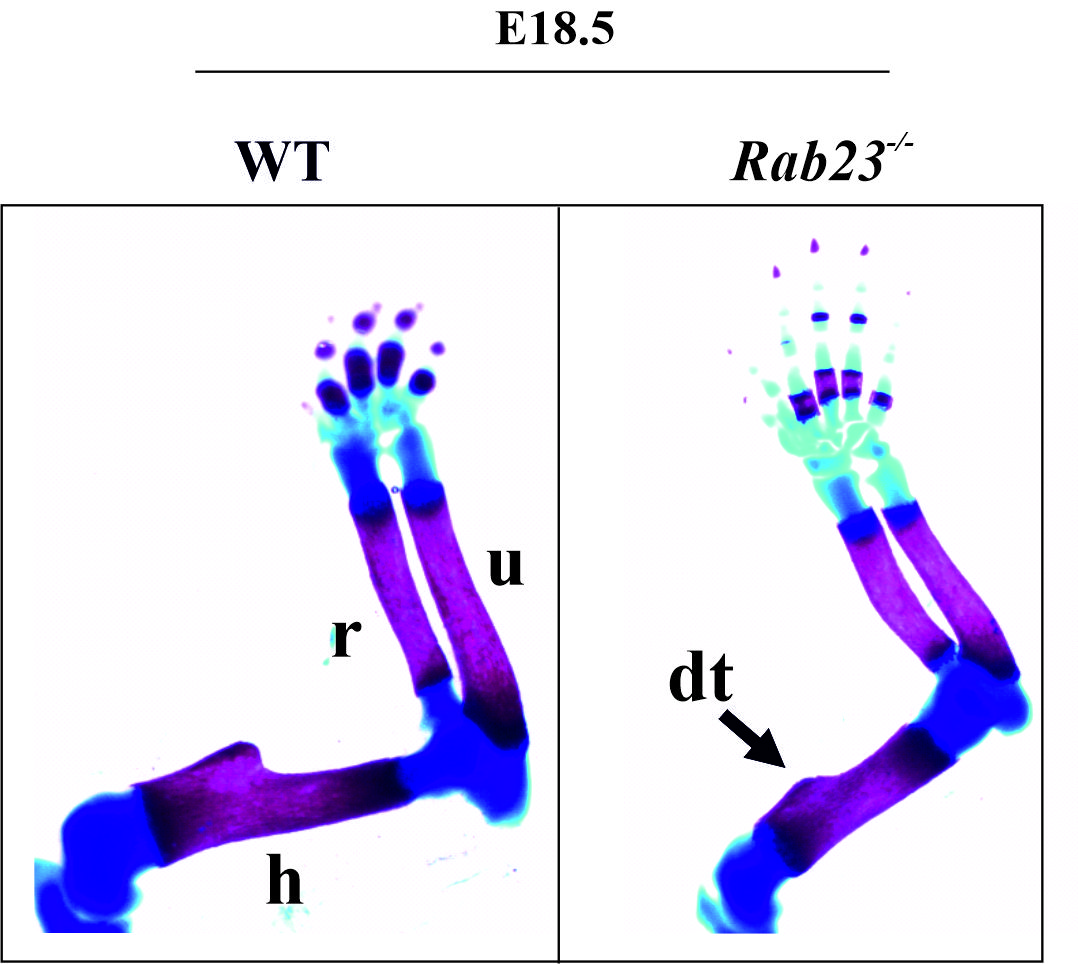

Supplement: Supplementary file 5 [file Image5.JPEG]

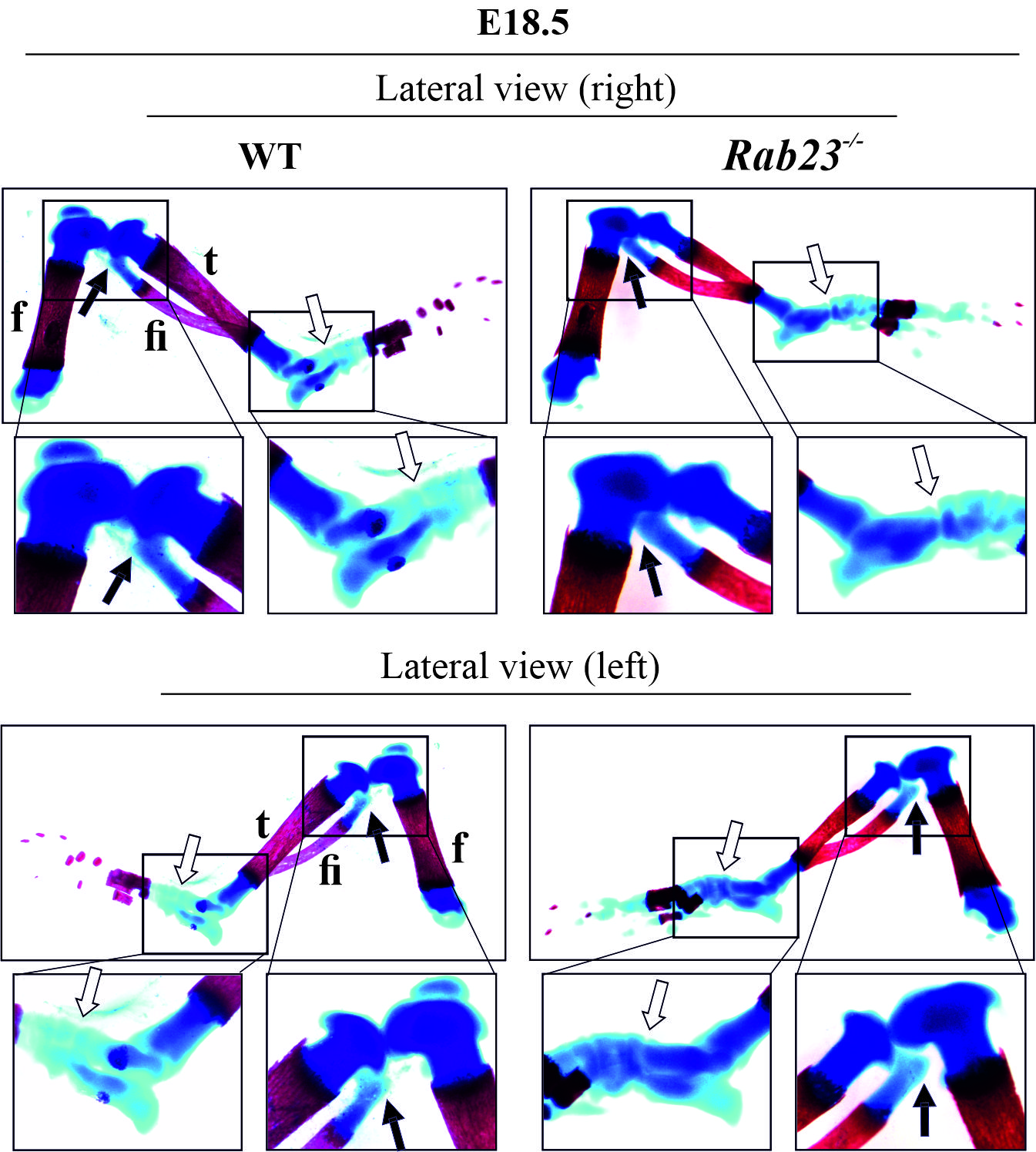

Supplement: Supplementary file 6 [file Image6.JPEG]
